# Research progress in extracorporeal shock wave therapy for upper limb spasticity after stroke

**DOI:** 10.3389/fneur.2023.1121026

**Published:** 2023-02-09

**Authors:** Haoyang Duan, Yawen Lian, Yuling Jing, Jingsong Xing, Zhenlan Li

**Affiliations:** Department of Rehabilitation Medicine, First Hospital of Jilin University, Changchun, China

**Keywords:** extracorporeal shock wave therapy, spasticity, post-stroke, progress, existing problems

## Abstract

Spasticity is one of the most common complications after stroke. With the gradual intensification of spasticity, stroke patients will have a series of problems such as joint ankylosis and movement restriction, which affect the daily activities and increase the burden on patients' families, medical staff and society. There are many ways to treat post-stroke spasticity before, including physical therapy and exercise therapy, drug therapy, surgery and so on, but not satisfied because of a few shortcomings. In recent years, many researchers have applied extracorporeal shock wave therapy (ESWT) for the treatment of post-stroke spasm and achieved good clinical effect, because it is non-invasive, safe, easy to operate, low cost and other advantages compared with other treatment methods. This article reviews the research progress and existing problems of ESWT in the treatment of post-stroke spasticity.

## 1. Introduction

Stroke, also known as “stroke” and “cerebrovascular accident” (CVA), is an acute cerebrovascular disease, which is a group of diseases caused by the sudden rupture of blood vessels in the brain or the blockage of blood vessels that prevents blood from flowing, causing brain tissue damage (1). Stroke is the third leading cause of human death, and its incidence is second only to tumor and ischemic heart disease. Stroke is also one of the leading causes of long-term disability in humans (2), which survivors are often accompanied by varying degrees of dyskinesia, sensory impairment, cognitive impairment, speech disorders and swallowing disorders and other sequelae (3, 4).

Especially noticed spasticity is one of the most common complications in stroke patients and usually occurs during the recovery period of stroke, after 3 months following stroke (5). About 4–42.6% of stroke patients experience spasticity after stroke onset, and 2–13% of these patients may cause severe disability (6, 7). Early in stroke, the presence of a spastic state means that the patient's muscle tone improves and motor function recovers; however, as the spastic state gradually increases, the patient may experience problems such as pain, pressure sores, muscle weakness, joint ankylosis, and limitation of movement (8–10), leading to difficulties in daily activities and increasing the burden on the patient's family, medical staff, and society. The cost of medical care increases nearly a four-fold in patients with stroke in the presence of spasticity compared with patients without spasticity (11).

In 1980, Lance first defined spasticity as “a movement disorder characterized by velocity-dependent hypertonia due to stretch hyperreflexia, and mostly associated with tendon hyperreflexia, as a form of upper motor neuron syndrome” (12), which is widely used and has greatly facilitated advances in the research and management of spasticity. Young and so on combined possible mechanisms of spasticity after stroke to define spasticity as “a movement disorder characterized by velocity-dependent tonic stretch hyperreflexia due to abnormal processing of primary inputs within the spinal cord” (13). Other definitions are descriptive, such as “hypertonia, which is characterized by resistance to externally imposed movements that increases with the rate of stretching and changes with the direction of joint movement,” “sensorimotor dysregulation caused by lesions of upper motor neurons, which manifests as intermittent or persistent involuntary activation of muscles” and “spasticity is involuntary muscle hyperactivity that occurs with central paralysis” (14–16). Due to the complexity and diversity of symptoms of post-stroke spasticity, there is no uniform definition of spasticity in the international academic so far. Regarding the definition of spasticity after stroke, we more recommend Lance's description of the definition of spasticity. First, spasticity must be one of the manifestations of upper motor neuron syndrome. The increased muscle tension without upper motor neuron system damage cannot be described as “spasticity,” which is one of the mistakes that many people are prone to make. Second, the spasticity is a velocity-dependent hyperreflexia of stretch reflex, which is the theoretical basis for our correct evaluation of spasticity—rapid stretch.

Moderate spasm in stroke patients can reduce the occurrence of complications, such as prevention of osteoporosis, prevention of venous thrombosis, a certain degree of muscle tension can also help maintain muscle capacity, slow down the degree and speed of muscle atrophy, and spasm of knee extensor muscle groups can assist patients to achieve standing and transfer (17, 18). However, severe spasticity may lead to muscle contracture, abnormal posture, pain, joint deformity, tissue contracture, etc., causing stiffness and discomfort, affecting daily care, washing, dressing, perineal hygiene and normal sitting posture, resulting in limited activities of daily living and decreased quality of life (19–21). Therefore, it is necessary and important to treat severe spasticity for stroke patients in rehabilitation.

There are currently many treatments for spasticity after stroke, including physical therapy and exercise therapy, drug therapy, and surgical treatment (22). Among them, physical therapy has the advantages of safety, cheapness, non-invasiveness, and effectiveness, and is one of the most commonly used methods for the treatment of spasticity, and is generally used as an adjunct to other treatments, including vibration therapy, transcutaneous electrical nerve stimulation, neuromuscular electrical stimulation, transcranial direct current stimulation, repetitive transcranial magnetic stimulation, biofeedback therapy, caloric stimulation therapy, orthosis use, good limb position placement, proprioceptive stimulation, stretching therapy, and movement therapy (23–26). Drug therapy is the main way to treat spasm, of which oral drugs used to treat systemic spasm affecting multiple sites include baclofen, diazepam, dantrolene and tizanidine; while for the treatment of small range spasm, local injection of botulinum toxin or ethanol and phenol block are mostly used (27–30). Because of the complexity of surgical operation and the limitation of therapeutic effect, surgical treatment is rarely used in clinical practice. It is mainly used for relieving severe spasm, correcting deformity and contracture, and preventing and treating complications when drug therapy and other interventions cannot effectively relieve spasm and the patient's activities of daily living and functional status are seriously affected. In fact, due to the side effects of drug therapy, invasiveness of local therapy, and economic burden, spasm sometimes cannot be effectively controlled even with many treatment modalities, so it is necessary to find new, economical, non-invasive, and effective treatment modalities to relieve spasm and improve the quality of life of patients. In recent years, many scholars have used extracorporeal shock wave therapy (ESWT) to treat spasticity after stroke and achieved good clinical results (31, 32). Because it has the advantages of non-invasive, safe, easy operation, and low cost compared with other treatment methods, more and more scholars have conducted in-depth studies. This article reviews the research progress of ESWT in the treatment of upper limb spasticity after stroke, in order to provide the basis for ESWT in the treatment of post-stroke upper limb spasticity.

## 2. Mechanism of spasticity after stroke

Regarding the occurrence of spasticity, at present, the mechanism of spasticity after stroke was divided into two categories: hypertonia due to stretch reflex, that is, reflexively mediated mechanism; hypertonia due to soft tissue changes, that is, non-reflexively mediated mechanism (33).

With regard to the reflex mediated mechanism of spasticity, neurophysiology currently believes that spasticity is the result of enhanced stretch reflex (12). In stroke patients with spastic hemiplegia, on the one hand, motor cortex and its corticospinal descending tract are injured, which will immediately lead to hemiplegia after stroke, mainly including upper and lower limb muscles on the affected side, and trunk muscles are also affected to some extent (34). On the other hand, due to the damage of the cortical bulbar pathway accompanied by the damage of the motor cortex or the corticospinal descending tract, the supraspinal inhibition was lost, and bulbar spinal cord overexcitation occurred (35). This is mainly a de-inhibition phenomenon, or de-masking effect. Reticulocyte hyperexcitability provides unopposed excitatory descending inputs to spinal extension reflex circuits, resulting in increased excitability of spinal motor neurons (36). This adaptive change can explain most of the clinical manifestations of spasticity, such as hyperreflexia, velocity dependent resistance to stretch, excessive muscle activity, or spontaneous discharge of motor units (37–39).

In addition to hyperreflexia, spasticity after stroke is also associated with changes in the mechanical properties of muscles, tendons, joints and other tissues, that is, non-reflex factor-mediated muscle spasm mechanism (33). Spasticity after stroke is mostly attributed to brain injury itself and less attention is paid to muscle tissue structure, metabolism and function. Muscle function and structure are altered following spasm, mainly by changes in muscle fiber size and fiber type distribution; extracellular matrix proliferation as measured morphologically and biochemically; and increased stiffness of spastic muscle cells (40–42). Spastic muscle has poorer mechanical properties of extracellular material compared to normal muscle (43, 44). In conclusion, the changes of muscle fiber classification, proportion and length of spastic muscles after stroke will promote the changes of physiological function and biomechanical characteristics of the affected skeletal muscles, and these changes further aggravate the spastic state.

## 3. Introduction to ESWT

ESWT is a series of single-pulse high-energy mechanical waves, characterized by high pressure (100 MPa), rapid pressure increase (< 10 ns), and short action cycle (10 μs), which can propagate in three-dimensional space, and the propagation speed accelerates with the increase of pressure (45). It can be divided into two types: focused extracorporeal shock wave therapy (fESWT) and radial extracorporeal shock wave therapy (rESWT) (46). Focused shock wave concentrates acoustic wave energy on a certain focal zone on target tissue, with very high energy at focal zone. According to different pressure generating devices, it can be divided into electrohydraulic extracorporeal shock wave, electromagnetic extracorporeal shock wave and piezoelectric extracorporeal shock wave, with penetration depth up to 12 cm. X-ray or ultrasound-guided precise localization of treatment site is often required during treatment to avoid damage to surrounding tissues (47, 48). Dissipative shock waves transmit acoustic energy into the body in a radially attenuated manner through contact with the skin, and there is only one pneumatic ballistic type, which requires the coupling agent to be applied to the surface of the treatment site during treatment, which has a much shallower penetration depth of about 3.5 cm compared with focused extracorporeal shock waves, and has low energy and slow transmission speed. The treatment head can move flexibly during the operation, has a larger therapeutic range, does not require the use of other equipment for positioning, is safer, less expensive, and is more suitable for the treatment of soft tissues such as muscles and ligaments (49, 50).

At present, extracorporeal shock wave therapy is widely used in the treatment of spasticity after stroke, doctors will select focused or dissipated extracorporeal shock waves according to the actual situation of patients themselves. Therefore, the adverse reactions of patients during the treatment period were few and mild, well tolerated, and the treatment could be repeated (51, 52).

## 4. Mechanisms of ESWT in treating spasticity after stroke

Currently, with the depth study on the mechanism of extracorporeal shock wave therapy for spasticity after stroke, it believed that there may be the following mechanisms.

### 4.1. ESWT induced nitric oxide synthesis

Nitric oxide (NO) is a non-classical neurotransmitter that acts as a messenger molecule in humans and is involved in neuromuscular junction formation as well as important physiological functions of the central nervous system, including the transmission, storage, and synapse-related functions of information in the nervous system (53, 54). Some scholars have proposed that extracorporeal shock waves can induce NO production through enzymatic and non-enzymatic pathways, reduce acetylcholine at the neuromuscular junction, and thus relieve muscle spasm (55, 56). Gotte et al. found that nitrite concentrations increased with increasing energy levels after shock waves applied to mixed solutions containing L-arginine and H_2_O_2_, providing a rationale for non-enzymatic pathways promoting NO production (57). Ciampa et al. found that shock waves increased the activity of nitric oxide synthase in the cytoplasm of rat glial cells C6 when shock waves were applied, thereby accelerating the production of NO. It also increases neuronal nitric oxide (nNOS) activity and NO production induced by a mixture of lipopolysaccharides (LPS), tumor necrosis factor-α (TNF-α), and interferon gamma (IFN-γ) (58). It has also been suggested that shock waves may temporarily reduce acetylcholine receptors at the neuromuscular junction, thereby affecting neuromuscular junction function and thereby reducing spasticity (56, 59). Shock waves can regulate NO synthesis and affect enzyme activity, so it is speculated that ESWT alleviates limb spasticity after stroke may be related to the regulation of NO synthesis.

### 4.2. Extracorporeal shock waves reduce motor neuron excitability

Some scholars believe that due to cerebral cortex damage after stroke, upper motor neurons lose their inhibitory effect on lower motor neurons, resulting in abnormal excitation of motor neurons (60, 61). Extracorporeal shock waves may reduce the excitability of motor neurons by vibratory stimulation of tendons, thereby achieving the goal of reducing tension. Leone et al. performed shock wave therapy on the Achilles tendon of hemiplegic patients to assess motor neuron excitability innervating the muscle by H-reflex and showed a decrease in H-reflex amplitude, suggesting that improving the functional status of the contractured Achilles tendon by continuous or intermittent compression of the Achilles tendon site may be related to regulating motor neuron excitability, but its neurophysiological effects and clinical effects could not be sustained (62). Daliri et al. observed the changes in the ratio of the maximum amplitude of H wave to the maximum amplitude of M wave (Hmax/Mmax value) after ESWT for flexor carpi spasm after stroke and found that the Hmax/Mmax value decreased, indicating that extracorporeal shock waves could improve the excitability of α motor neurons and thus relieve limb spasm (63).

### 4.3. ESWT modulates the process of nerve conduction

The occurrence of limb spasticity after stroke is not only related to the high excitability of motor neurons, but also related to the innervation and nerve conduction of muscles. Extracorporeal shock waves can produce nerve block at free nerve endings, neuromuscular junctions and other parts, thus regulating the conduction of nerves (64, 65). Ohtofi et al. used shock waves to act on rats, and all epidermal sensory nerve fibers degenerated within 1 week, and epidermal nerves regenerated after 2 weeks (66). Kenmoku et al. applied an extracorporeal shock wave to the right calf muscle of rabbits and found that there was degeneration of acetylcholine receptors in the muscle after shock wave application, causing transient nerve conduction dysfunction at the neuromuscular junction, suggesting that ESWT may cause transient dysfunction of nerve conduction, which in turn affects the functional status of the muscle, but the number of receptors that were reduced at the neuromuscular junction by shock wave application would recover at an extremely rapid rate, which may be the reason why shock wave treatment of spasticity is generally of short duration (67).

### 4.4. Effect of extracorporeal shock waves on muscle and soft tissue

Extracorporeal shock waves, acting on human tissues, may produce different physical stress effects in the area of action through the human medium, namely tensile stress and shear force, thereby causing tissue release, improving the microcirculation of muscles and thus relieving the state of muscle spasm in patients (68–70). A meta-analysis showed that ESWT improves passive joint mobility and muscle properties such as stiffness, tension, and elasticity, which are thought to be related to the rheological properties of shock waves on spastic muscles (71). Kim et al. found that after 2 weeks of ESWT, the range of motion significantly increased, and supraspinatus muscle strength, thickness, tension and stiffness were effectively restored (72). Lee et al. showed that ESWT helps revascularization by applying ESWT to lesions and reduces pain and improves function by stimulating or reactivating the healing process of connective tissue, including tendons and bones (73).

We believe that the mechanism of extracorporeal shock wave in treating spasticity after stroke is not single, but the result of multiple mechanisms. For different patients, a certain mechanism may be the main one but this needs further in-depth study.

## 5. Clinical study of ESWT in the treatment of upper limb spasticity after stroke

Most upper limb spasms after stroke are mainly flexor spasms, which seriously affect rehabilitation and hinder the improve of motor function. Severe spasms may lead to shoulder pain, shoulder hand syndrome, etc. In 2005, Manganotti et al. applied extracorporeal shock wave to patients with spastic stroke for the first time, and observed the effect of ESWT on upper limb dystonia in patients with stroke. They selected 20 stroke patients with hypermyotonia of the upper limbs, and gave 1,500 pulse shocks to the belly of the forearm flexor muscle and 3,200 pulse shocks to the hand interosseous muscle. The energy density was 0.030 mJ/mm^2^. The results showed that the muscular tension of flexor carpi and flexor digitorum could be effectively reduced immediately after ESWT compared with that before treatment. Among them, the flexor muscle tension of the finger was significantly improved 1 week, 4 weeks and 12 weeks after treatment compared with that before treatment. The flexor carpi muscle tension was significantly improved after 1 and 4 weeks of treatment compared with that before treatment, while it was not significantly improved after 12 weeks. In addition, the study measured the motor nerve conduction velocity and F wave response of the abductor digiti minimus muscle endings, and observed the shock wave stimulation of the ulnar nerve. The results showed that there was no indication of denervation changes such as potential changes, which failed to explain its mechanism. This study shows that ESWT can significantly reduce upper limb muscle spasm in stroke patients, especially for finger flexor muscle tension, and can maintain the effect for 12 weeks. During the study, patients had not reported any side effects related to extracorporeal shock waves (74). Kim et al. treated 57 stroke patients with subscapularis spasm with five times of divergent extracorporeal shock wave therapy for 2 weeks to observe whether this method is equally effective for subscapularis spasm. The researchers were followed up for 6 weeks before and after each shock wave treatment, evaluated once a week, and evaluated 11 times in total. The results confirmed that the rESWT could relieve the subscapular muscle spasm, relieve shoulder pain, and improve shoulder joint range of motion in patients with stroke. There is a significant difference between the evaluation indexes 4 weeks after treatment and those before treatment, and there is no significant difference between the indexes 6 weeks after treatment, but the overall trend is still improved compared with those before treatment (75).

In recent years, more and more clinical research showed that ESWT can reduce the upper limb flexor muscle tension of stroke patients and effectively improve muscle spasm (76–78). Troncati et al. observed the ESWT of 12 hemiplegic patients with upper limb spasticity. The energy density of the abdominals of forearm flexors was 0.105 mJ/mm^2^ for 1,600 pulses, and the energy density of hand interosseous muscles was 0.08 mJ/mm^2^ for 800 pulses. The results showed that the muscular tension of rotator cuff, elbow flexor, wrist flexor and finger flexor decreased after treatment. After 3 and 6 months of treatment, the muscular tension of rotator cuff, flexor carpi and flexor digitorum decreased compared with that before treatment, while the muscular tension of elbow flexor did not change significantly compared with that before treatment. The passive range of motion of upper limb joints was significantly improved after treatment, 3 months after treatment and 6 months after treatment. It is believed that shock wave therapy has a definite effect on upper limb motor function, and can significantly improve upper limb motor function, which can last for 6 months. This study shows that ESWT has a long-term effect on upper limb spasm in stroke patients (79). Li et al., in order to further observe the effective time of ESWT in relieving spasticity, performed rESWT treatment on the antagonist and agonist muscles of the upper limb elbow in stroke patients. The results showed that rESWT could improve spasticity and pain by acting on spasmodic antagonists or agonists. The therapeutic effect lasted at least 4 weeks, but it could not improve the motor function of upper limbs (80). Park et al. randomly divided 30 stroke patients into the treatment group and the control group. MyotonPrO muscle detector was used to detect the upper limb muscle tension of stroke patients after ESWT treatment. The results showed that the mechanical indexes of flexor carpi ulnaris, flexor carpi radialis and flexor digitorum in the treatment group were significantly higher than those in the control group, suggesting that ESWT can effectively relieve the upper limb flexor spasm (81).

Some scholars focus on muscle spasms of small joints of upper limbs such as wrist joint, metacarpophalangeal joint, etc. Gjerakaroska Savevska et al. reported that a 42 year old ischemic stroke patient was treated with ESWT for his right hand spasm. After six times of rESW treatment, the efficacy was evaluated according to the modified Ashworth scale (MAS) and disability rating scale (DAS) scores before treatment, after the sixth treatment, and 1 and 3 months after the end of treatment respectively. The results showed that ESW could improve the spasticity of wrist and finger flexors after stroke, and the effect was continuous (82). Daliri et al. observed 15 patients with wrist flexor spasm after stroke one time of comfort treatment and one time of routine ESWT 1 week later, and recorded the changes of motor function at each stage in the MAS score, Hmax/Mmax value and Brunstrom evaluation method. The results showed that the MAS scores and Hmax/Mmax values after conventional ESWT treatment were improved compared with those after placebo treatment, but no significant changes in motor function were observed in each stage of Brunstrom assessment. It shows that single ESWT treatment can relieve wrist flexor spasm after stroke, but has no significant improvement in motor function (63).

To sum up, ESWT treatment can better relieve upper limb spasticity after stroke, and last the effect for a long time. ESWT treatment is effective not only for typical flexor spasms, but also for agonists and antagonists. At present, ESWT is used in the treatment of upper limb spasticity after stroke, and the treatment intensity mostly is selected low energy as the treatment intensity. Although there are two types of shock wave and the treatment methods include fESWT and rESWT. There is no significant difference have shown in the therapeutic effect between them these two extracorporeal shock waves.

## 6. Current problems

The current clinical research shows that the main adverse reaction of ESWT in the treatment of muscle spasticity in stroke patients is pain and ecchymosis in the local part of the action site, but these can be disappeared in a short time (83–85). As the known literature has not been followed up for a long time, there may also be long-term side effects, but they have not been found yet. In addition, there is no unified reference standard for the treatment parameters, site, case selection, evaluation scale selection, etc. of shock wave treatment of spastic state. Most of the existing clinical studies are based on the experience of clinicians, lacking objectivity. The clinical treatment scheme of shock wave needs further exploration.

### 6.1. Treatment site and treatment parameters

Yoon et al. compared the efficacy of extracorporeal shock wave in the treatment of limb spasticity in different parts of stroke patients. MAS score and modified Tardieu scale (MTS) score were the main evaluation indicators. The results showed that the effect of extracorporeal shock wave on muscle abdomen or the junction between muscle abdomen and tendon was better (86). Our recommended treatment sites are to include the muscle belly, the tendon symphysis, and the tendon, which is the whole muscle portion of the target muscle. Troncati et al. showed that the MAS scores of wrist flexor, finger flexor and shoulder external rotator muscles decreased after 3 and 6 months of treatment, but there was no significant change in elbow flexor muscle. It was believed that the lack of sample size might be due to factors such as different muscle locations, insufficient shock wave dose and intensity. On the other hand, in clinical research, the treatment time and follow-up time are inconsistent, so it is difficult to determine the best treatment interval of EWST (79). At present, there is no uniform standard for the therapeutic parameters of ESWT treatment for spasticity, including the intensity, frequency, number of pulse, duration of session, which needs more in-depth clinical research.

### 6.2. Case selection

A meta-analysis by Mihai et al. (87) shows that ESWT has long-term clinical efficacy in relieving lower limb spasticity, reducing pain intensity and increasing range of activity in stroke patients, but Bae et al. found that EWST has immediate efficacy (88). It may be that the inclusion criteria of Bae et al. include patients with mild spasticity, which is not enough to achieve lasting effects. Another reason may be that the patient has a long course of disease, muscle spasm lasts for a long time, and periarticular tissue sclerosis lasts for a long time. Only repeated shock wave treatment can alleviate the stiffness of these connective tissues. We suggest that the criteria for inclusion should be more strictly specified in future studies to make the research results referential and to exclude false results caused by non-standard inclusion criteria.

### 6.3. Selection of evaluation scale

It is a problem worthy of discussion to evaluate the curative effect of muscle spasm with a high credit rating scale. At present, MAS has a high reliability (89, 90) in the clinical evaluation scale of muscle spasm, but MAS does not take into account the speed dependent components of muscle spasm and the degree of muscle flexion and extension when resistance occurs. According to the definition of Lance spasticity (12), MAS cannot fully assess the degree of spasticity. MTS reflects the speed dependence of spasticity by comparing the resistance of muscles during passive stretching at slow speed and fast speed, so it is considered to be more suitable for Lance spasticity assessment than MAS (91, 92). On the other hand, the MAS score did not include clinical symptoms such as pain, convulsions and effects on function, which are closely related to muscle spasm. On the contrary, clinical symptoms such as twitching, pain and decreased ability of daily living may occur with muscle spasm. Therefore, other clinical scales such as visual analysis scale (VAS), modified Barthel index and Fugl Meyer evaluation scale (93–95) should be used to observe the therapeutic effect of ESWT.

## 7. Conclusion and future outlook

Recent studies have confirmed that ESWT can safely, economically and effectively treat upper limb spasticity after stroke, reduce the use of antispasmodic drugs in patients with upper limb spasticity after stroke, and thus reduce adverse drug reactions. The side effects observed so far are mainly tolerable pain and ecchymosis, but these can disappear quickly. However, at present, ESWT still has some limitations: ① There are few basic researches on ESWT, and its mechanism is still unclear. ② There is no uniform standard for the treatment parameters of ESWT, and the best parameters of ESWT for muscle spasm cannot be determined. ③ The duration of the effect of ESWT on post-stroke spasticity is still unclear. Therefore, the mechanism of action, treatment parameters and duration of efficacy of ESWT are the key directions of future research, providing more basis for its further clinical promotion, and making its application in patients with upper limb spasms after stroke more standardized.

## Author contributions

All authors listed have made a substantial, direct, and intellectual contribution to the work and approved it for publication.
